# Agenesis of the left hepatic lobe undergoing laparoscopic hepatectomy for hepatocellular carcinoma: a case report

**DOI:** 10.1186/s40792-017-0325-z

**Published:** 2017-03-29

**Authors:** Katsunori Matsushita, Kunihito Gotoh, Hidetoshi Eguchi, Yoshihumi Iwagami, Daisaku Yamada, Tadafumi Asaoka, Takehiro Noda, Hiroshi Wada, Koichi Kawamoto, Yuichiro Doki, Masaki Mori

**Affiliations:** Department of Gastroenterological Surgery, Graduate School of Medicine, Osaka University, 2-2-E2, Yamadaoka, Suita, Osaka 565-0871 Japan

**Keywords:** Agenesis, Liver, Left lobe, Hepatocellular carcinoma

## Abstract

**Background:**

Agenesis of the left hepatic lobe is a rare anomaly. It is defined as the absence of liver tissue to the left of the gallbladder fossa. Additionally, agenesis of the left hepatic lobe accompanied by hepatocellular carcinoma is quite rare. We experienced the case of a patient with agenesis of the left hepatic lobe, undergoing laparoscopic hepatectomy for HCC.

**Case presentation:**

A 79-year-old man was referred to our department with epigastralgia. Abdominal computed tomography revealed agenesis of the left hepatic lobe, accompanied by hepatocellular carcinoma in segments 7 and 8. He underwent laparoscopic partial hepatectomy of segments 7 and 8. The operative findings revealed complete agenesis of the liver to the left of the falciform ligament. The patient had a favorable clinical course without liver dysfunction or any complications.

**Conclusions:**

We experienced a case with agenesis of the left hepatic lobe undergoing laparoscopic hepatectomy for HCC. Awareness of such anomaly is important for surgeons to avoid postoperative complications.

## Background

Agenesis of a hepatic lobe is defined as the absence of liver tissue to the right or left of the gallbladder fossa [1]. The incidence of hepatic lobe agenesis is very low [2, 3]. Because agenesis of a hepatic lobe is clinically asymptomatic, it is usually an incidental finding, revealed by imaging examinations [4]. We report the case of a patient with agenesis of the left hepatic lobe, who underwent laparoscopic hepatectomy for hepatocellular carcinoma (HCC).

## Case presentation

### Case

**Fig. 1:**
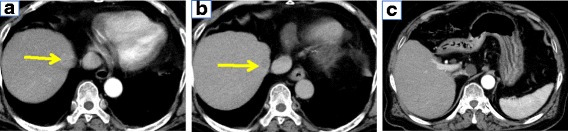
CT shows the agenesis of the left hepatic lobe accompanied by HCC. **a** Arterial phase of dynamic CT shows a 15-mm hypervascular mass in segment 8. **b** Delayed phase shows a hypovascular mass. **c** Abdominal CT shows the nonexistence of the left hepatic lobe

A 79-year-old man with a history of myelodysplastic syndrome presented to the Department of Hematology in our hospital. He was referred to our department with epigastralgia. He had no history of blood transfusion. And he had no smoking and drinking history. Laboratory data were as follows: white blood cell 12,460/μL, red blood cell 513 × 10^4^/μL, hemoglobin 16.1 g/dL, platelet 15.3 × 10^4^/μL, total bilirubin 0.8 mg/dL, aspartate aminotransferase 50 U/L, alanine aminotransferase 62 U/L, alkaline phosphatase 303 U/L, γ-glutamyltransferase 199 U/L, albumin 4.2 g/dL, prothrombin time 98.0%. Tests for hepatitis B virus surface antigen and antibodies against hepatitis C virus were negative. His Child-Pugh classification was class A. His serum alpha-fetoprotein level was 254 ng/mL, and the serum levels of CA19-9, CEA, and proteins induced by the absence of vitamin K were within the normal range. Abdominal CT revealed a 15 mm high attenuation with early-phase enhancement and late-phase washout in segment 8 (Fig. 1a, b). He was diagnosed with HCC.

**Fig. 2:**
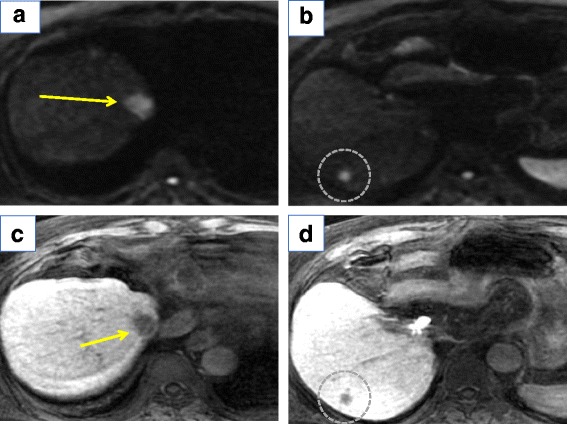
Gd-EOB-DTPA-enhanced MRI shows two lesions, one each in segments 7 and 8. **a, b** Diffusion-weighted imaging showing high-intensity lesions. **c**, **d** Gd-EOB-DTPA-enhanced MRI shows low-intensity lesions in the hepatocellular phase

**Fig. 3:**
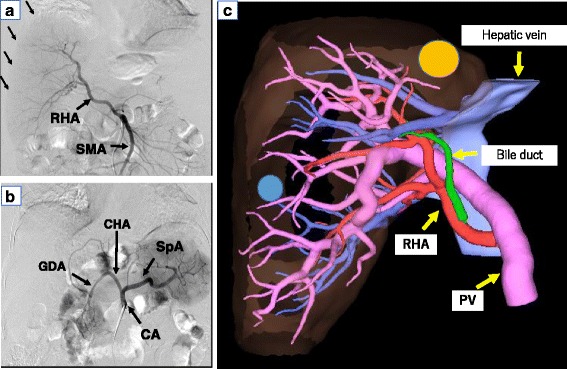
Abdominal angiography and MPR images show agenesis of the left hepatic lobe. **a** Superior mesenteric arteriography shows the right hepatic lobe was visualized. **b** Celiac arteriography shows no region of the liver was visualized. **c** MPR image shows the absence of the left hepatic artery, left portal vein, and left hepatic biliary system. *Circle zone* (*yellow*) shows the lesion of S8. *Circle zone* (*blue*) shows the lesion of S7

Computed tomography (CT) findings revealed the absence of the left lobe of the liver (Fig. 1c). Magnetic resonance imaging (MRI) showed a 15-mm nodule in segment 8 (S8) and an 8-mm nodule in segment 7 (S7). Both tumors showed hyperintensity on diffusion-weighted imaging (Fig. 2a, b) and hypointensity on the hepatocellular phase (Fig. 2c, d). Superior mesenteric angiography showed the right hepatic artery and right hepatic lobe (Fig. 3a), but celiac angiography showed no region of the liver (Fig. 3b). Three-dimensional CT showed the absence of the left hepatic artery, left portal vein, and left hepatic biliary system (Fig. 3c).

**Fig. 4:**
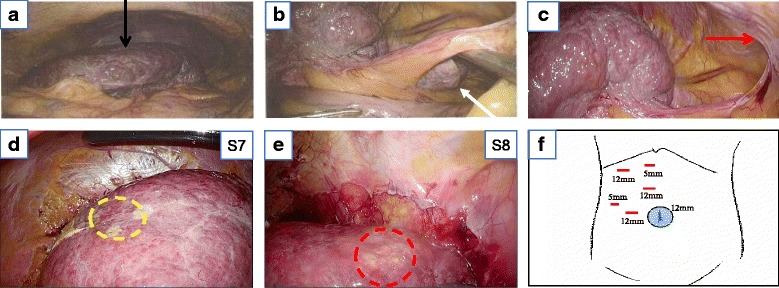
Intraoperative findings show the absence of the left hepatic lobe. **a** *Arrow* (*black*) shows the entire liver. The gross appearance of the liver is nodular cirrhosis. **b** *Arrow* (*white*) shows no hypertrophy of the caudate lobe. **c** This image shows complete agenesis of the liver to the left of the falciform ligament (*red arrow*). **d** *Circle* (*yellow*) shows the lesion of S7. **e** *Circle* (*red*) shows the lesion of S8. **f** The schema demonstrates the placement of each port

Based on the radiological findings, our preoperative diagnosis was HCC in S7 and S8, accompanied with agenesis of the left hepatic lobe. The clinical stage of HCC was T2N0M0, stage II, in terms of the Union for International Cancer Control classification (seventh edition) [5]. Intraoperative findings showed absence of the left lobe of the liver (Fig. 4a). There was no hypertrophy of the caudate lobe or of the right hepatic lobe (Fig. 4b). There was complete agenesis of the liver to the left of the falciform ligament (Fig. 4c). These findings confirmed the diagnosis of congenital agenesis of the left hepatic lobe. The right hepatic lobe had cirrhosis. Tumors were located near the surface of the liver in this case. Therefore, we performed laparoscopic partial hepatectomy for S7 and S8.

**Fig. 5:**
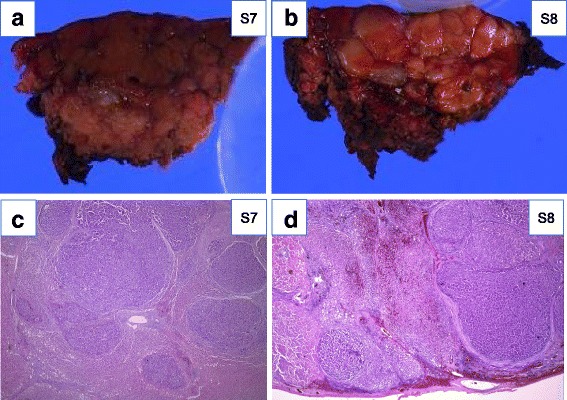
Macroscopic findings and histological examination of the resected specimen of segments 7 and 8. **a** The specimen of S7 demonstrates confluent multinodular type, 14 mm in diameter. The weight of the specimen was 10 g. **b** The specimen of S8 demonstrates confluent multinodular type, 4.5 mm in diameter. The weight of the specimen was 15 g. **c** The lesion of S7 demonstrates moderately differentiated hepatocellular carcinoma (H&E staining ×20). **d** The lesion of S8 demonstrates poorly differentiated hepatocellular carcinoma (H&E staining ×20)

Macroscopically, the specimen resected from S7 and S8 appeared to be confluent multinodular type, 14 and 4.5 mm in diameter, respectively. Histological examination of the tumors showed moderately differentiated (S7) and poorly differentiated (S8) HCC (Fig. 5a, b). The non-cancerous portion of the resected liver confirmed a diagnosis of liver cirrhosis. The patient had a favorable clinical course without liver dysfunction or any complications, and he was discharged on postoperative day 11.

### Discussion

Agenesis of the hepatic lobe is defined as the absence of liver tissue to the right or left of the gallbladder fossa [1]. Congenital absence of the left hepatic lobe is a rare anomaly [6]. It is defined as a congenital disorder of the hepatic lobe, and it was first reported by Arnold and Ashley-Montagu in 1932 [7]. The incidence of lobar agenesis has been reported to be 0.005% in 19,000 autopsy cases [3].

We experienced a patient with agenesis of the left hepatic lobe who also had hepatocellular carcinoma. We reviewed 34 cases, including our case, of agenesis of the left hepatic lobe reported in the literature. The patients included 23 men and 11 women aged 41–78 years at diagnosis, with a mean age of 62 years. In previous reports, seven cases had malignant comorbidities, four cases of gastric cancers, one case of hepatocellular carcinoma, and two cases of other malignant diseases [8, 9]. In the literature, a possible relationship between agenesis of the left hepatic lobe and malignant comorbidities was not discussed in detail.

Agenesis of the liver is clinically asymptomatic with normal liver function [10, 11]. In this case, abdominal CT incidentally revealed agenesis of the left hepatic lobe during an examination for epigastralgia. No structure could be recognized as the left hepatic artery, left hepatic vein, or the portal vein. There was no hypertrophy of the caudate lobe or of the right hepatic lobe. Imaging studies revealed agenesis of the left hepatic lobe. He underwent laparoscopic partial hepatectomy of S7 and S8. The operative findings confirmed complete agenesis of the liver to the left of the falciform ligament.

The congenital factors associated with hypoplasia of the hepatic lobe are an anomaly of the umbilical vein and abnormal development or thrombosis of the portal venous segment during embryologic growth [12]. Failure to establish a bloodstream to the liver is important in the agenesis of the hepatic lobe. Acquired factors associated with bloodstream blockage to the liver include liver cirrhosis, vascular occlusion by a tumor, and Budd-Chiari syndrome [8].

It is important to eliminate the possibility of atrophy of the liver before making a diagnosis of agenesis of the liver. Benz and Baggenstoss listed some reasons for atrophy of the liver as obstruction of the bile ducts, stenosis of the portal veins and their branches, pressure on the left portal vein by dilatation of bile ducts, and severe malnutrition [13]. In this case, we cannot deny the possibility that the agenesis of the left hepatic lobe was caused by cirrhosis. However, the tumors themselves did not cause the agenesis of the hepatic lobe because of no major vascular invasion. Moreover, there was no deformation or compensatory hypertrophy of the right hepatic lobe or caudate lobe. In addition, the agenesis of the left hepatic lobe in our patient was noted when he underwent cholecystectomy 30 years ago. Therefore, in our case, the diagnosis was congenital agenesis of the left hepatic lobe.

Agenesis of the left lobe of the liver is usually associated with digestive disorders such as gastric ulcer, sigmoid volvulus, and displaced duodenum as a result of fixed defects of the digestive system and an abnormal course of the biliary system [14]. The abnormal hepatic morphologic characteristics lead to displacement of the gallbladder, resulting in compression and torsion of the cystic duct. It may lead to the complications of severe iatrogenic biliary injuries. In our case, we did not identify any digestive disorders and abnormal biliary system. Pre-surgical knowledge of such anatomical alterations is necessary for surgical planning to avoid fatal surgical complications.

Four (11%) of 34 cases including our case were accompanied by cirrhosis [8]. There is no known mechanistic relationship between the incidence of cirrhosis and agenesis of the left hepatic lobe. Most cases of agenesis of the left hepatic lobe have normal liver function. Two (6%) of 34 cases including our case were accompanied by HCC. Thus, its incidence seems to be low in these patients.

Hepatectomy is a standard treatment for HCC [15]. Minamoto et al. reported agenesis of the left hepatic lobe in a patient undergoing posterior segmentectomy for hepatocellular carcinoma. They reported the patient’s postoperative clinical course was favorable, similar to our case, along with restoration of the resected hepatic volume about 1 year after surgery [9]. Although accurate preoperative evaluation of the tumor status and liver function reserve is necessary for preventing postoperative liver failure [16], hepatectomy is acceptable for a HCC patient with agenesis of the left hepatic lobe.

## Conclusions

We experienced a case with agenesis of the left hepatic lobe undergoing laparoscopic hepatectomy for HCC. Awareness of such anomaly is important for surgeons to avoid postoperative complications.

## Abbreviations

AFP: Alpha-fetoprotein
ALP: Alkaline phosphatase
ALT: Alanine aminotransferase
AST: Aspartate aminotransferase
CA: Celiac artery
CA-19-9: Carbohydrate antigen 19–9
CBD: Common bile duct
CEA: Carcinoembryonic antigen
CHA: Common hepatic artery
CT: Computed tomography
GDA: Gastroduodenal artery
GTP: γ-Glutamyl transpeptidase
HBs-Ag: Hepatitis B virus surface antigen
HCC: Hepatocellular carcinoma
HCV-Ab: Hepatitis C virus antibody
K PIVKA-II: Protein induced by vitamin K absence or antagonist II protein induced by the absence of vitamin
MRI: Magnetic resonance imaging
PV: Portal vein
RHA: Right hepatic artery
RHV: Right hepatic vein
S: Segment
SMA: Superior mesenteric artery
SpA: Splenic artery
